# Supplementary material for: Sex-dependent rescue of memory and synaptic deficits in AD model mice by increasing PSD-95 palmitoylation
Source: Commun Biol. 2026 Feb 18;9:451. doi: 10.1038/s42003-026-09702-y (PMC13031330; doi:10.1038/s42003-026-09702-y)
Supplement: Supplementary file 2 — Supplementary Information [file 42003_2026_9702_MOESM2_ESM.pdf]

Supplementary information for

Sex-dependent rescue of memory and synaptic deficits in AD model mice by increasing PSD-95 palmitoylation

Yixing Du[1], Katie Prinkey[1], Andrew Q. Pham[1*], Amber Lawrence[1*], Celeste Morales[1*], Maureen Dinata[1*], Marlenne Gutierrez[1*], Ahmed Khalil[1*], Medha Sharma[2], Robert A. Rissman[3], Mehreen Manikkoth[1], Ian Baick[1], Haritha Karthikeyan[1] and Kim Dore[1#]

1-Department of Neurosciences, University of California San Diego, La Jolla, CA 92093, USA

2-Department of Pharmacology, University of California San Diego, La Jolla, CA, 92093, USA

3-University of Southern California, Keck School of Medicine, Alzheimer's Therapeutic Research Institute, San Diego, CA, 92121, USA

* - These authors contributed equally

#- Corresponding author: kdore@ucsd.edu

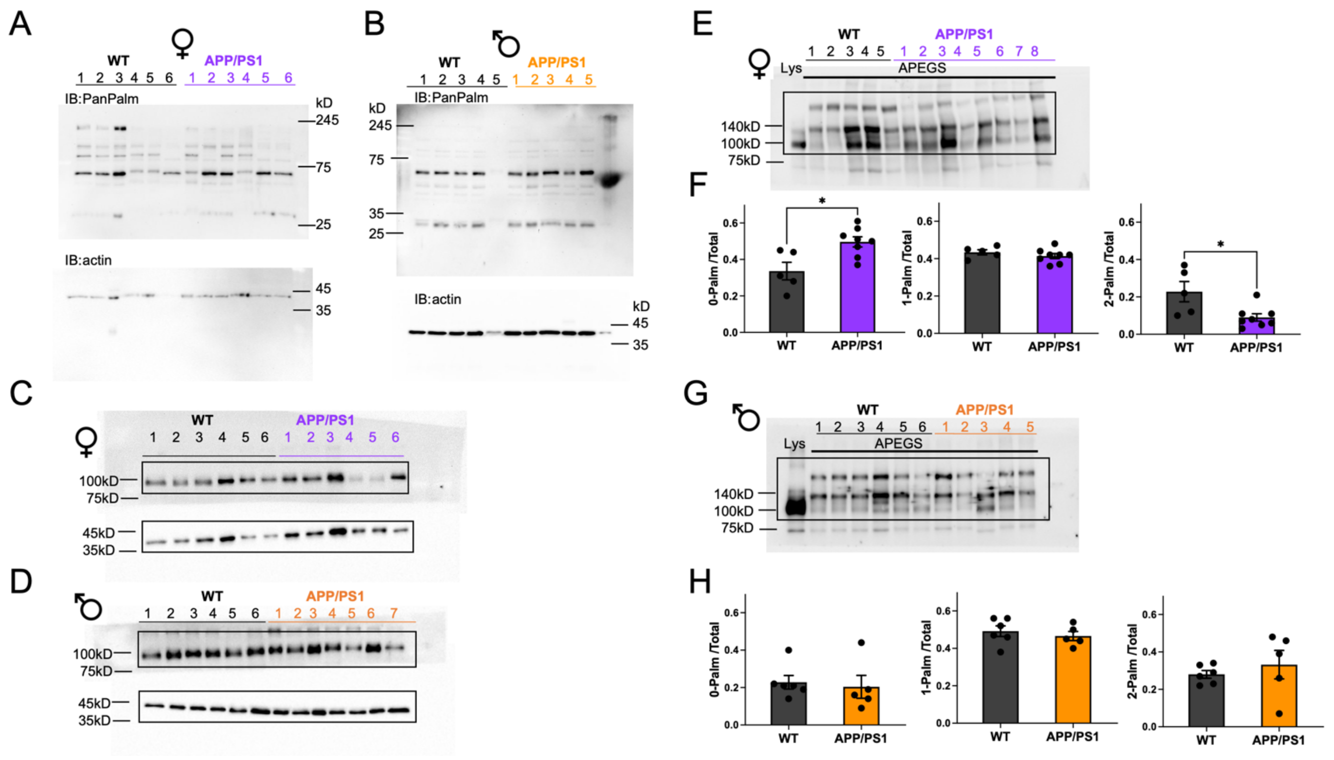

Supplementary Figure 1: Total protein palmitoylation, PSD-95 levels and PSD-95 palmitoylation in female and male WT and APP/PS1 mice

A) Uncropped Western Blot of total protein palmitoylation in female mice (see Fig. 1A).

B) Uncropped Western Blot of total protein palmitoylation in male mice (see Fig. 1B).

C) Uncropped Western blots of hippocampal lysates from female mice. Immunoblotting with PSD-95 and actin. (see Fig. 1E-F).

D) Uncropped Western blots of hippocampal lysates from male mice. Immunoblotting with PSD-95 and actin. (see Fig. 1G-H).

E) Uncropped Western Blot of hippocampal lysates from female mice that underwent the APEGS assay (see Fig. 1I).

F) Quantification of the ratio of the 0-Palm band (left), the 1-Palm band (middle) and the 2-Palm band (right) over the sum of the three bands (total PSD-95) in the Western Blot shown in E). N=5 WT and N=8 APP/PS1 mice. * $p<0.05$, Mann Whitney test.

G) Uncropped Western Blot of hippocampal lysates from male mice that underwent the APEGS assay (see Fig. 1K).

H) Quantification of the ratio of the 0-Palm band (left), the 1-Palm band (middle) and the 2-Palm band (right) over the sum of the three bands (total PSD-95) in the Western Blot shown in G). N=6 WT and N=5 APP/PS1 mice. Not significant, Mann Whitney test.

Error bars in all panels represent +/- SEM.

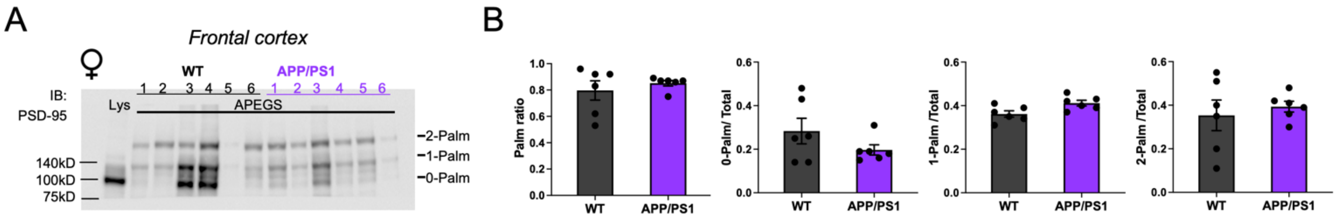

Supplementary Figure 2: PSD-95 palmitoylation is not affected in the frontal cortex of female APP/PS1 mice

A) Uncropped Western Blot of frontal cortex lysates from female mice that underwent the APEGS assay.

B) Quantification of the Palm ratio (1st graph), the 0-Palm band (2nd graph), the 1-Palm band (3rd graph) and the 2-Palm band (4th graph) over the sum of the three bands (total PSD-95) in the Western Blot shown in A). N=6 WT and N=6 APP/PS1 mice. Not significant, Mann Whitney test. Error bars represent +/- SEM.

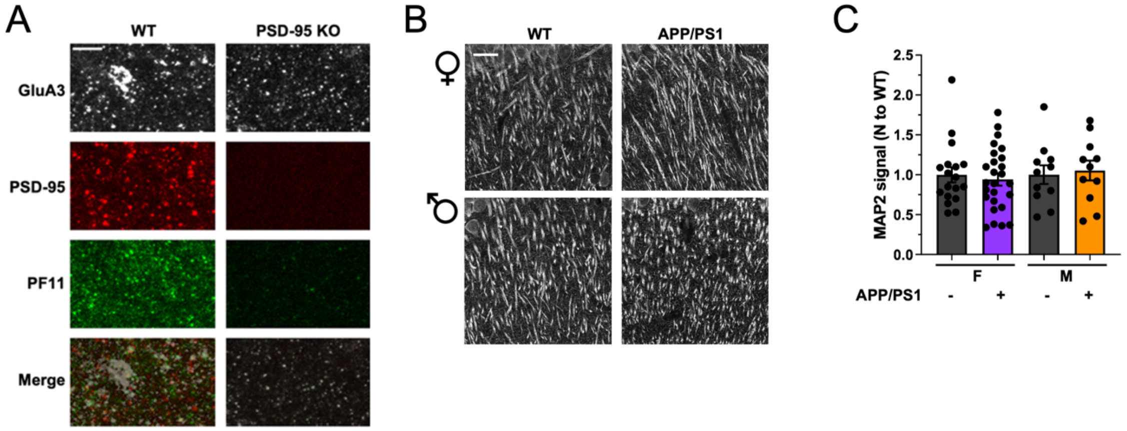

Supplementary Figure 3: Measurement of PSD-95 and PF11 signals with IHC in mouse brain slices

A) IHC for GluA3, PSD-95 and PF11 in WT and PSD-95 KO mice in the CA1 region of the hippocampus, scale bar is 5 µm.

B) IHC for MAP2 in the CA1 of WT and APP/PS1 mice of both sexes, scale bar is 10 µm.

C) Quantification of IHC shown in B, N=18 for WT Females, N=26 for APP/PS1 females, N=11 for WT males and APP/PS1 males. For all IHC data, N denotes the number of mice. Two-way ANOVA (F (3,36) = 0.165, p=0.919). Error bars represent +/- SEM

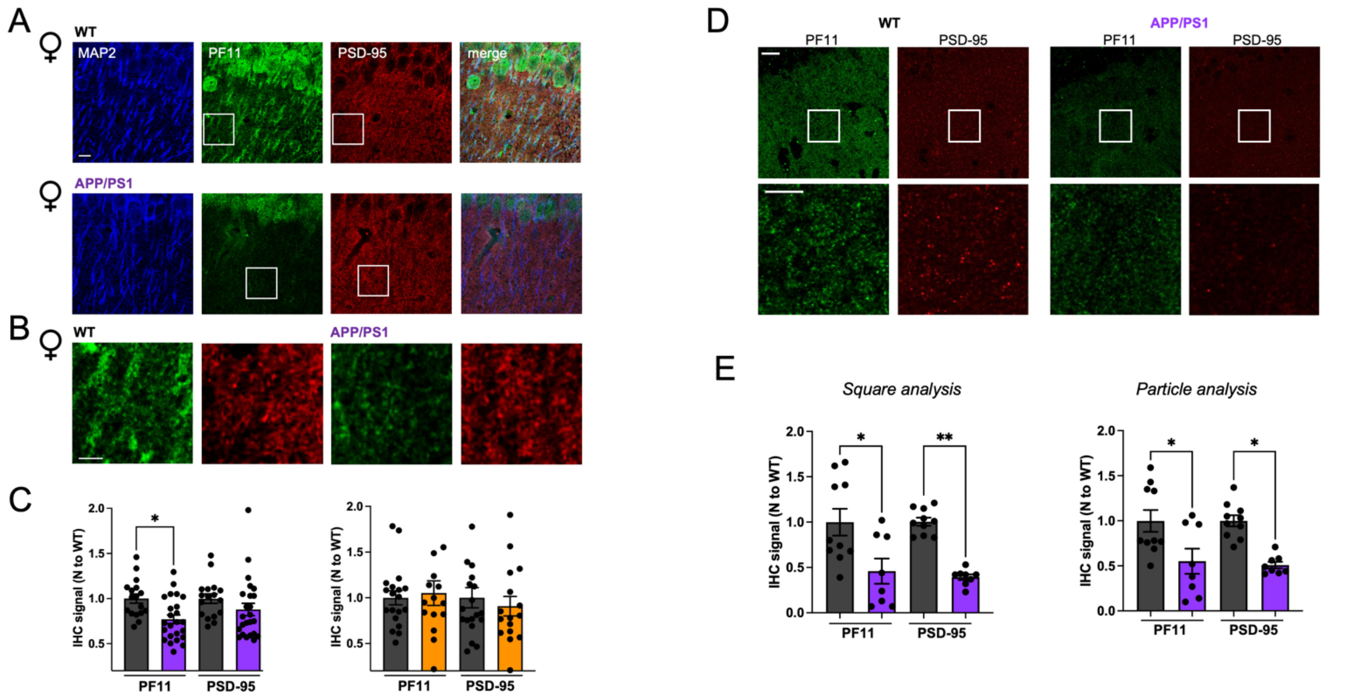

Supplementary Figure 4: IHC experiments show reduction in PF11 signal in female APP/PS1 mice

A) Representative examples of MAP2, PF11 and PSD-95 immunostaining in the CA1 region of the hippocampus in female mice. Scale bar is 10 µm. White box indicates region of interest shown in B).

B) Zoomed in images of PF11 and PSD-95 in WT and APP/PS1 female mice, Scale bar is 5µm.

C) Quantification of PF11 and PSD-95 signal in female (N=17 mice for WT, N=24 for APP/PS1) and male (N=19 mice for WT, N=15 for APP/PS1) mice. * $p < 0.05$, One-way ANOVA (effect of genotype, $F(3,78) = 3.66$ $p = 0.016$) followed by Šídák post-hoc test.

D) IHC for PF11 (green) and PSD-95 (red) in 8 µm thick slices in WT or APP/PS1 female mice, scale bar is 20 µm for top row images, and 10 µm for inset images shown below. Note absence of signal with the PF11 antibody in cell bodies. See Methods for details.

E) Quantification of IHC using square analysis (left, as in other figures) N=10 for WT and N=8 for APP/PS1 (slices). Two-way ANOVA ($F(3,23) = 6.804$, $p=0.0019$) followed by Tukey's post-hoc test, * $p < 0.05$, ** $p < 0.01$. Right, same data analyzed with particle analysis (see Methods); Two-way ANOVA ($F(3,23) = 6.338$, $p=0.0027$) followed by Tukey's post-hoc test, * $p < 0.05$.

Error bars in all panels represent +/- SEM

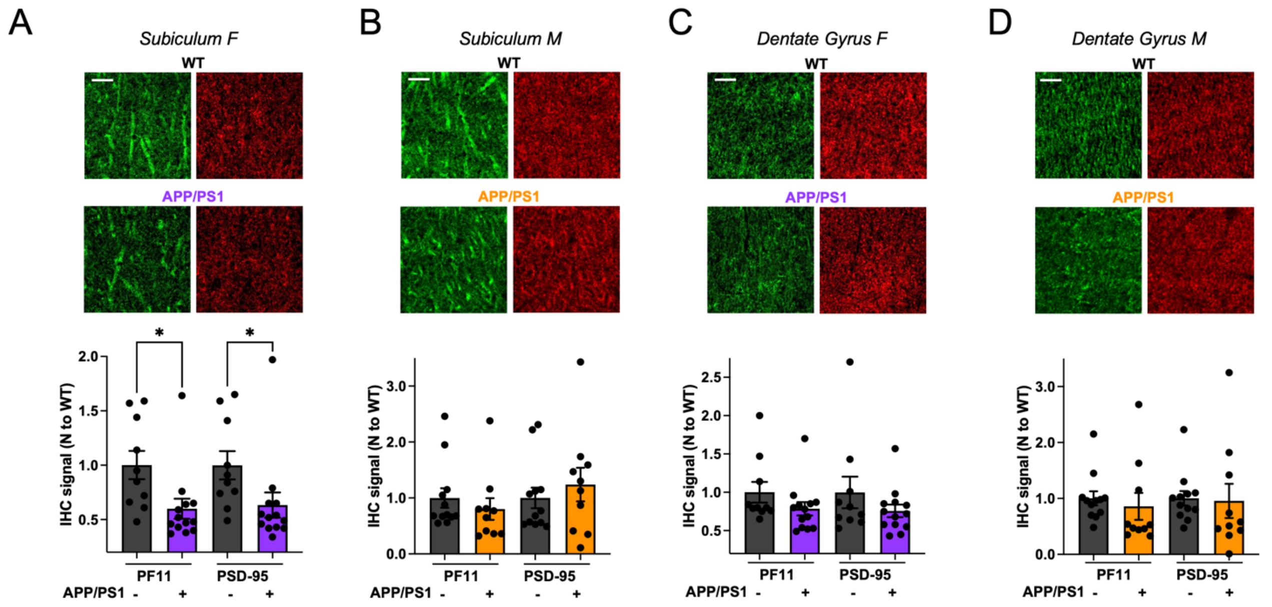

Supplementary Figure 5: Total PSD-95 and PF11 signals are also reduced in the subiculum of female APP/PS1 mice

A) Top: Representative images of PF11 (green) and PSD-95 (red) IHC in the subiculum of female mice, scale bar is 10 µm. Below: Quantification of IHC signal; N=10 for WT, N=13 for APP/PS1. Two-way ANOVA ($F(3,30) = 4.049$, $p=0.0157$) followed by Fisher LSD post-hoc test, * $p< 0.05$.

B) Top: Representative images of PF11 and PSD-95 IHC in the subiculum of male mice, scale bar is 10 µm. Below: Quantification of IHC signal; N=12 for WT, N=10 for APP/PS1. Two-way ANOVA ($F(3,29) = 0.679$, $p=0.57$).

C) Top: Representative images of PF11 and PSD-95 IHC in the dentate gyrus of female mice, scale bar is 10 µm. Below: Quantification of IHC signal; N=10 for WT, N=13 for APP/PS1. Two-way ANOVA ($F(3,30) = 1.422$, $p=0.26$).

D) Top: Representative images of PF11 and PSD-95 IHC in the dentate gyrus of male mice, scale bar is 10 µm. Below: Quantification of IHC signal; N=12 for WT, N=10 for APP/PS1. Two-way ANOVA ($F(3,29) = 0.304$, $p=0.82$).

Error bars in all panels represent +/- SEM

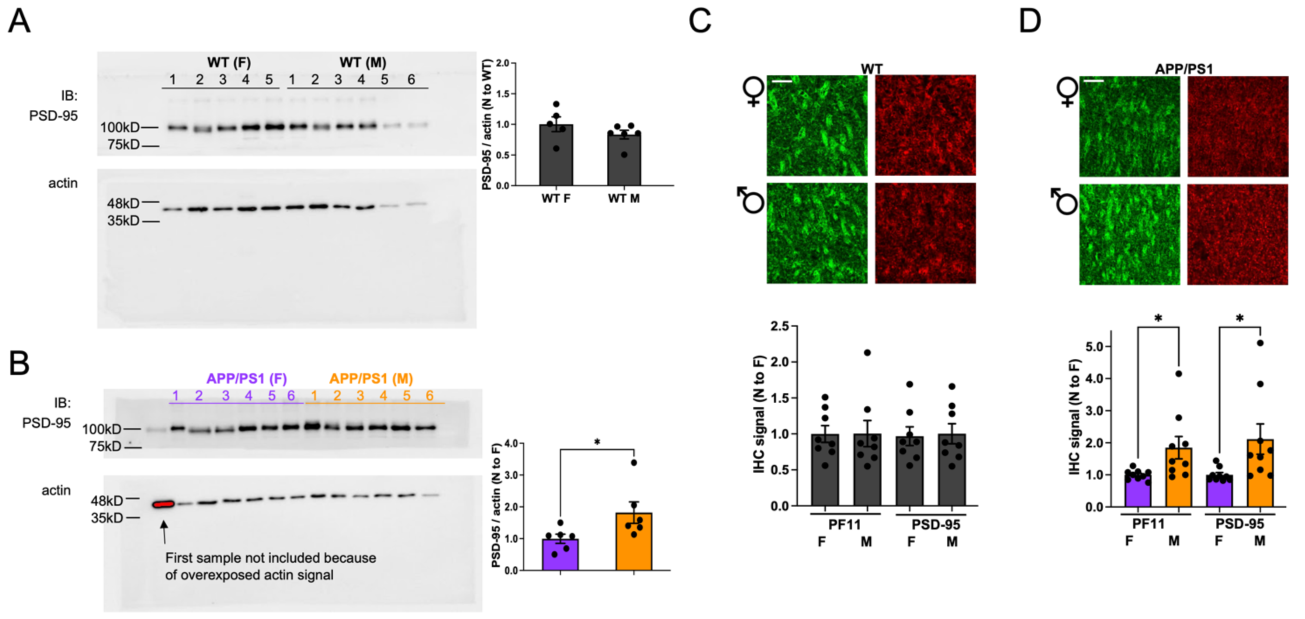

Supplementary Figure 6: Male APP/PS1 mice have more hippocampal PSD-95 than female APP/PS1 mice

A) Left: uncropped Western Blot showing no change in PSD-95 levels in WT mice. Right: quantification graph. N=5 WT males and N=6 WT females. Not significant, Unpaired T-test.

B) Left: uncropped Western Blot showing lower in PSD-95 in the hippocampus of APP/PS1 female mice. Right: quantification graph. N=9 APP/PS1 mice (males and females). * $p < 0.05$, Unpaired T-test. Note that the first lane was not included in the analysis because of the overexposed actin signal due to unequal loading.

C) Female and male WT mice have similar levels of PF11 signal (green) and total PSD-95 (in red), scale bar is 10 µm. N=8 WT males and females. Two-way ANOVA ($F(3,19) = 0.361$, $p=0.782$).

D) PF11 signal and total PSD-95 levels are lower in female APP/PS1 mice (N=9) than in male mice (N=9), scale bar is 10 µm. Two-way ANOVA ($F(3,32) = 3.78$, $p=0.02$) followed by Fisher LSD post-hoc test, * $p < 0.05$.

Error bars in all panels represent +/- SEM

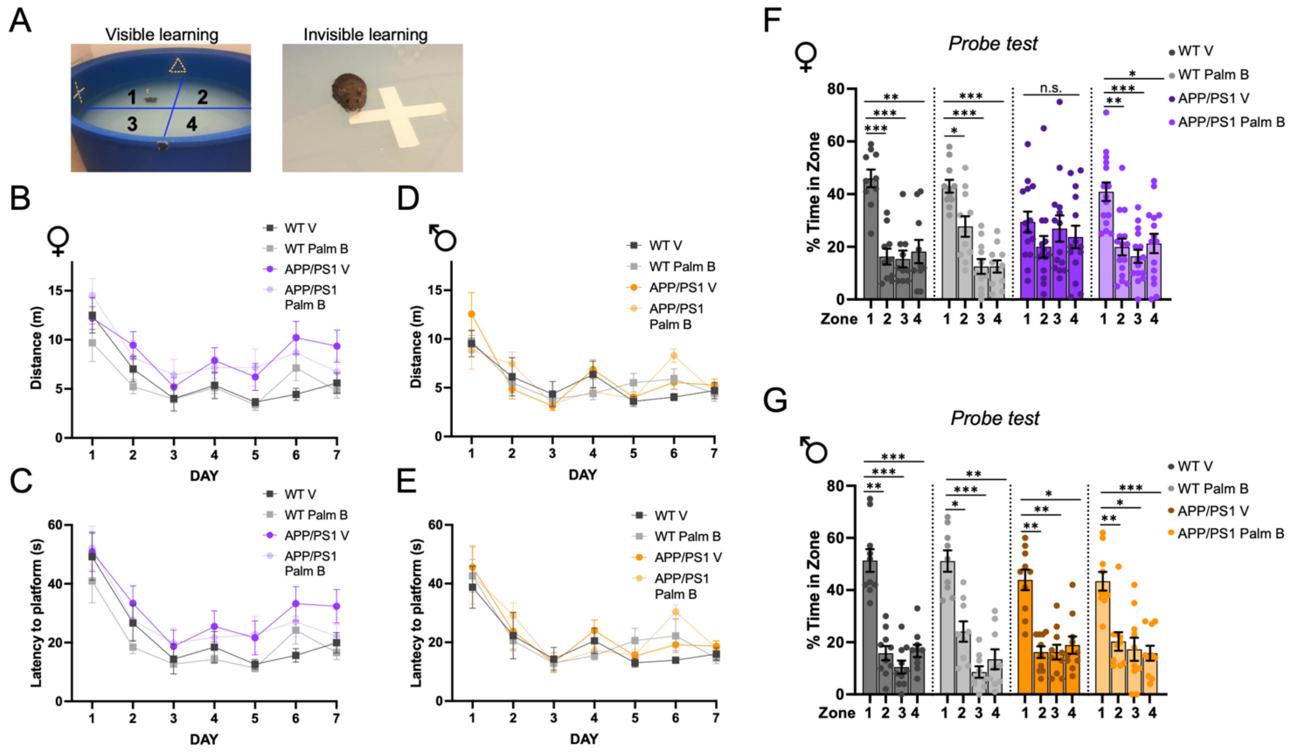

Supplementary Figure 7: Morris Water Maze behavioral testing

A) Picture of the Morris Water Maze tank with the 4 zones identified during visible learning (left), picture of a mouse on the submerged platform during invisible learning (right).

B) Average swimming distance during training trials for female mice. N=10 mice for WT (V), N=11 for WT (PB), N=14 for APP/PS1 (V), N=15 for APP/PS1 (PB).

C) Average latency to platform during training trials for female mice (same animals as in B).

D) Average swimming distance during training trials for male mice N=10 mice for WT (V), N=9 for WT (PB) N=10 for APP/PS1 (V), N=10 for APP/PS1 (PB).

E) Average latency to platform during training trials for male mice (same animals as in D).

F) % Time in each zone during the probe test for female mice (same animals as in B, C). One-way repeated measures ANOVA for each condition; $p < 0.001$ for WT V, WT Palm B and APP/PS1 Palm B, not significant for APP/PS1 V; followed by Dunnett's multiple comparisons test; ** $p < 0.01$, *** $p < 0.001$.

G) % Time in each zone during the probe test for male mice (same animals as in D, E). One-way repeated measures ANOVA for each condition; $p < 0.001$ for WT V, WT Palm B, APP/PS1 V and APP/PS1 Palm B; followed by Dunnett's multiple comparisons test; * $p < 0.05$, ** $p < 0.01$, *** $p < 0.001$.

Error bars in all panels represent +/- SEM

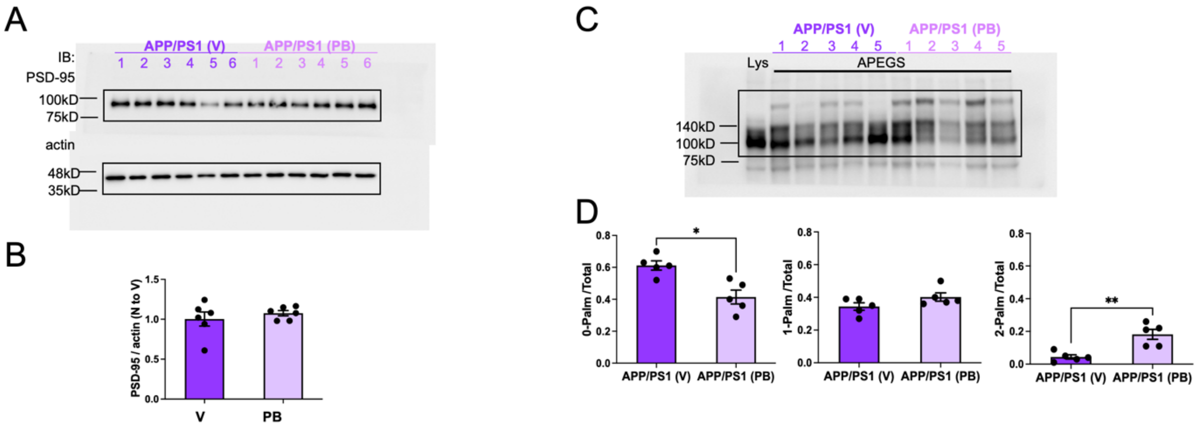

Supplementary Figure 8: Palm B injections increase PSD-95 palmitoylation in female APP/PS1 mice

A) Uncropped Western blot of hippocampal lysates from female APP/PS1 mice. Immunoblotting with PSD-95 and actin (see Fig. 2F).

B) Quantification of the Western Blot shown in A). Not significant, Mann Whitney test.

C) Uncropped Western Blot of hippocampal lysates from female APP/PS1 mice that underwent the APEGS assay (see Fig. 2H).

D) Quantification of the ratio of the 0-Palm band (left), the 1-Palm band (middle) and the 2-Palm band (right) over the sum of the three bands (total PSD-95) in the Western Blot shown in C). N=5 WT and APP/PS1 mice. * $p < 0.05$, ** $p < 0.01$, Mann Whitney test.

Error bars in all panels represent +/- SEM

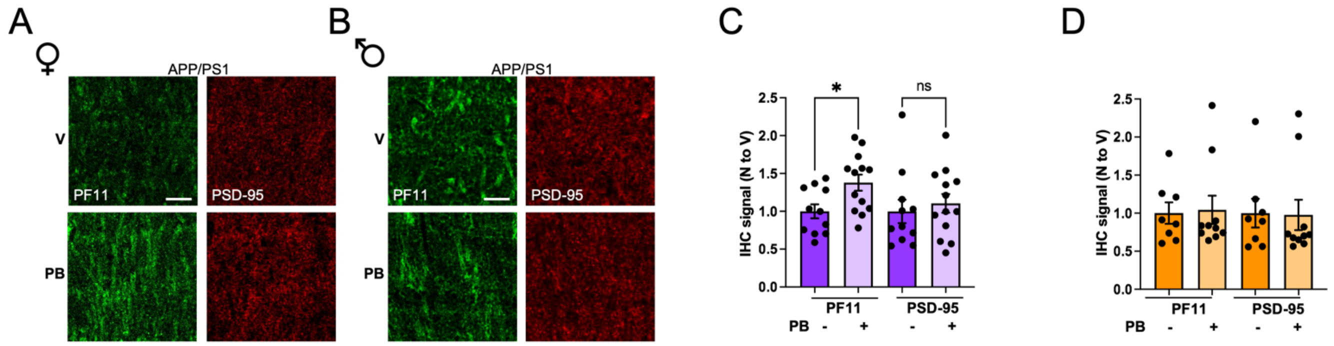

Supplementary Figure 9: Palm B injections increase PF11 signal in female APP/PS1 mice only

A) Representative images of PF11 (green) and PSD-95 (red) immunostaining in the CA1 of female APP/PS1 mice treated with vehicle (V), top or Palm B (PB), below. Scale bar is 10 µm.

B) Representative images of PF11 and PSD-95 immunostaining in the CA1 of male APP/PS1 mice treated with vehicle (V), top or Palm B (PB), below. Scale bar is 10 µm.

C) Quantification of PF11 and PSD-95 signal in female APP/PS1 mice. N=11 mice treated with vehicle (V) and N=13 for mice treated with Palm B (PB). One-way ANOVA (effect of treatment, $F_{(3,32)} = 3.3$ $p=0.029$) followed by Tukey post-hoc test. * $p < 0.05$.

D) Quantification of PF11 and PSD-95 signal in male APP/PS1 mice. N=8 mice for mice treated with vehicle (V) and N=10 for mice treated with Palm B (PB).

Error bars in all panels represent +/- SEM

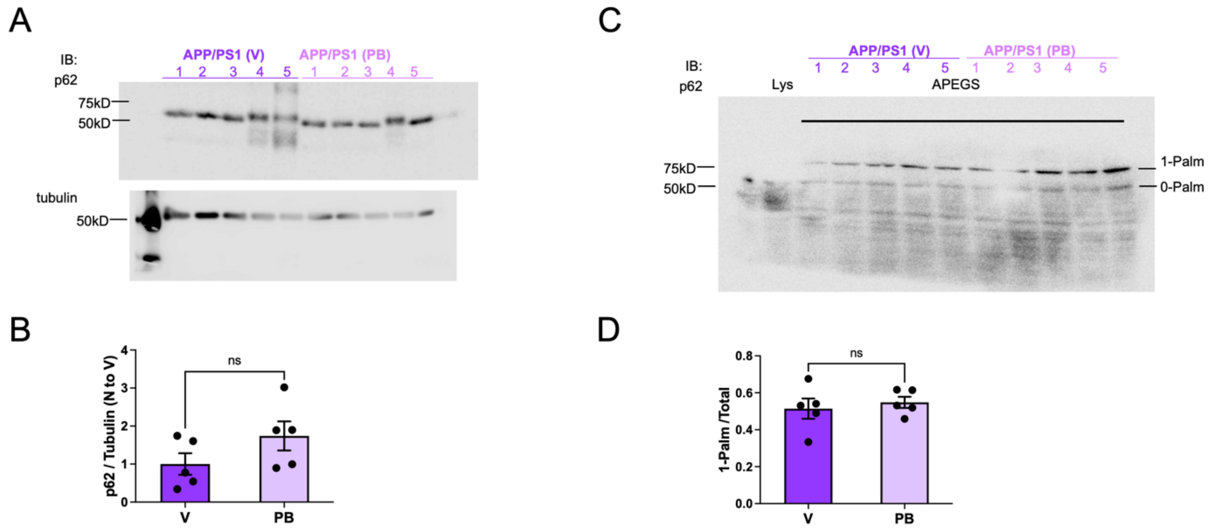

Supplementary Figure 10: Palm B does not affect hippocampal levels or palmitoylation of the APT1 target p62

A) Uncropped Western blot of hippocampal lysates from female APP/PS1 mice. Immunoblotting with p62 and tubulin.

B) Quantification of the Western Blot shown in A. N=5 for WT and APP/PS1 mice. Not significant, Mann Whitney test.

C) Uncropped Western Blot of hippocampal lysates from female APP/PS1 mice that underwent the APEGS assay, immunoblotting with p62.

D) Quantification of the Western Blot shown in C. N=5 for WT and APP/PS1 mice. Not significant, Mann Whitney test.

Error bars in all panels represent +/- SEM

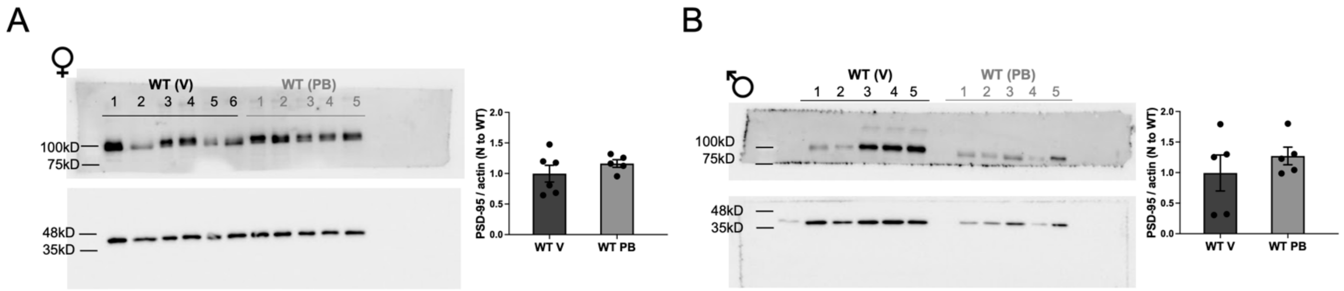

Supplementary Figure 11: Palm B has no effect on PSD-95 levels in WT mice

A) Left: uncropped Western blot of hippocampal lysates from WT female mice treated with vehicle (V) or Palm B (PB). Immunoblotting with PSD-95 and actin. Right: quantification of PSD-95 hippocampal levels. N= 6 mice for WT (V), N=5 for WT (PB). Not significant, Mann Whitney test.

B) Left: uncropped Western blot of hippocampal lysates from WT male mice. Immunoblotting with PSD-95 and actin. Right: quantification of PSD-95 hippocampal levels. N= 5 mice for WT (V) and WT (PB). Not significant, Mann Whitney test.

Error bars in all panels represent +/- SEM

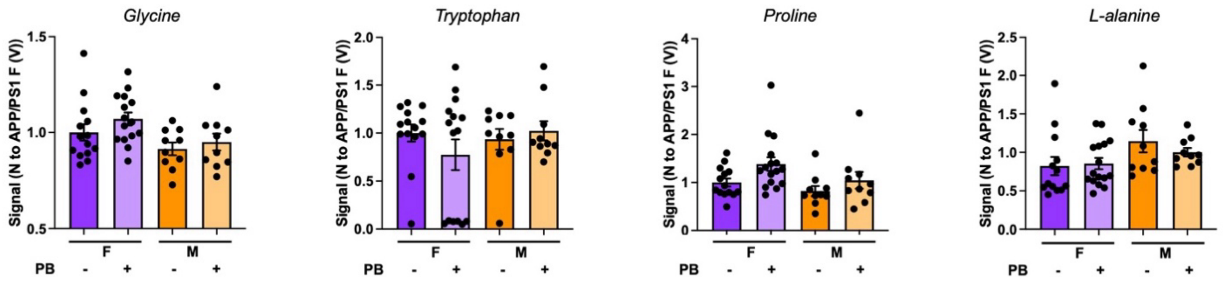

Supplementary Figure 12: Amino acids levels are similar in male and female APP/PS1 mice and are not affected by Palm B

For female mice, N=14 for APP/PS1 (V), N=15 for APP/PS1 (PB)). For male mice N=10 for APP/PS1 (V) and APP/PS1 (PB). Same samples as in Figure 3B. Two-way ANOVA (effect of sex and treatment) not significant for all four amino acids.

Error bars in all panels represent +/- SEM

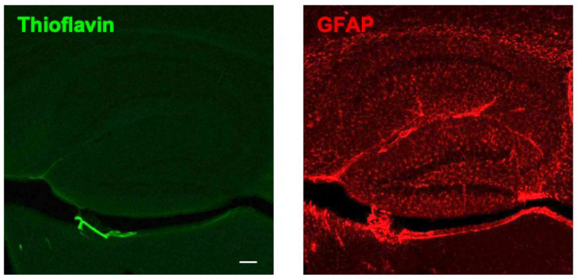

Supplementary Figure 13: GFAP IHC in the hippocampus of WT mice

Representative example of Thioflavin-S staining in WT mice, scale bar is 200μm (left); GFAP immunostaining in the same slice (right).

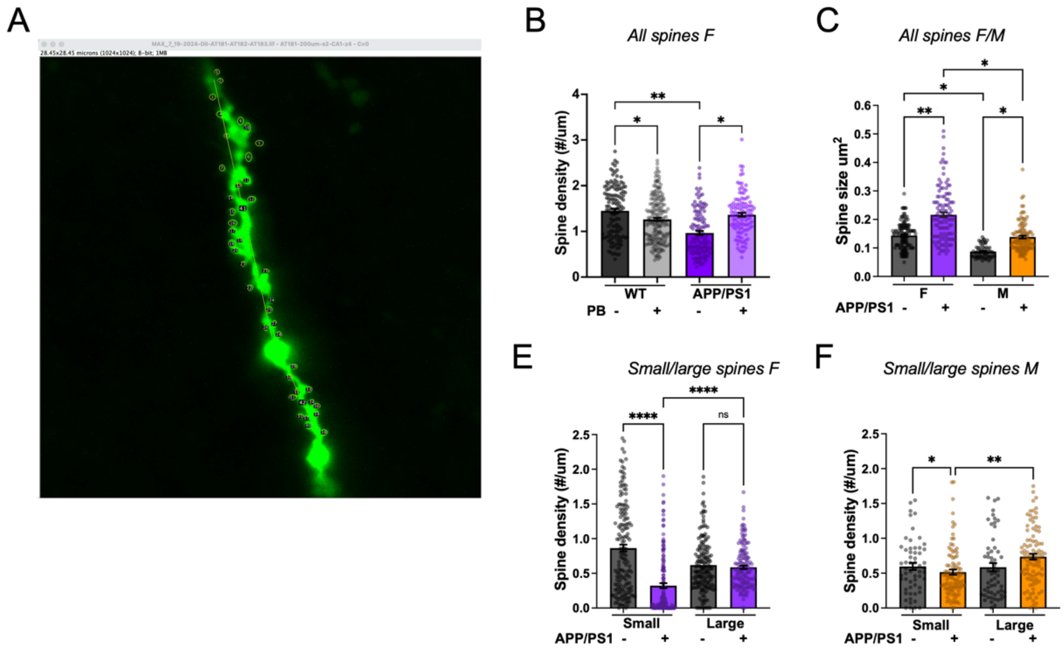

Supplementary Figure 14: Dendritic spine analysis in DiI stained CA1 dendrites in female and male mice

A) Representative image of DiI staining and spine analysis in WT female mice.

B) Spine density in all spines analyzed for female WT and APP/PS1 mice treated with vehicle or Palm B (PB). N=132 for WT (V), N=199 for WT (PB), N=120 for APP/PS1 (V), N=118 for APP/PS1 (PB); each data point is an individual dendrite section. Nested ANOVA, Type II Wald p-value of genotype:treatment = 0.00062 (see Methods); Tukey-adjusted pairwise post hoc comparisons, * $p < 0.05$, ** $p < 0.01$.

C) Spine size in all spines analyzed for female and male WT and APP/PS1 mice treated with vehicle. N=132 for WT F, N=21 for APP/PS1 F, N=59 for WT M, N=107 for APP/PS1 M. Nested ANOVA, Type II Wald p-value of genotype:sex = 0.0036; Tukey-adjusted pairwise post hoc comparisons, * $p < 0.05$, ** $p < 0.01$.

D) Spine density in all spines analyzed for female and male WT and APP/PS1 mice treated with vehicle (same dendrite sections as in C). Nested ANOVA, Type II Wald p-value of genotype:sex = 0.0056; Tukey-adjusted pairwise post hoc comparisons, * $p < 0.05$, ns, non-significant.

E) Spine density for female mice treated with vehicle sorted in small and large categories (based on median size of 0.13um^2). N=132 for WT, N=120 for APP/PS1. Two-way ANOVA (effect of genotype and size, $F(3,465) = 35.95$, $p < 0.0001$) followed by Tukey's post-hoc test. **** $p < 0.0001$.

F) Spine density for male mice treated with vehicle sorted in small and large categories (based on median size of 0.11um^2). N=59 for WT, N=107 for APP/PS1. Two-way ANOVA (effect of genotype and size, $F(3,208) = 5.175$, $p = 0.0018$) followed by Tukey's post-hoc test. * $p < 0.05$, ** $p < 0.01$.

Error bars in all panels represent +/- SEM
