# Supplementary material for: Sex-dependent rescue of memory and synaptic deficits in AD model mice by increasing PSD-95 palmitoylation
Source: Commun Biol. 2026 Feb 18;9:451. doi: 10.1038/s42003-026-09702-y (PMC13031330; doi:10.1038/s42003-026-09702-y)
Supplement: Supplementary file 3 — Description of Additional Supplementary File [file 42003_2026_9702_MOESM3_ESM.pdf]

Description of Additional Supplementary Files

File name: Supplementary Data 1

Description: Source data behind the graphs in Figures 1, 2 and 4

File name: Supplementary Data 2

Description: Source data behind the graphs in Figure 3

File name: Supplementary Data 3

Description: Source data behind the graphs in Figure 5
