# Supplementary material for: Sex-dependent rescue of memory and synaptic deficits in AD model mice by increasing PSD-95 palmitoylation
Source: Commun Biol. 2026 Feb 18;9:451. doi: 10.1038/s42003-026-09702-y (PMC13031330; doi:10.1038/s42003-026-09702-y)
Supplement: Supplementary file 7 — Reporting Summary [file 42003_2026_9702_MOESM7_ESM.pdf]

nature portfolio

Corresponding author(s): Kim Dore

Last updated by author(s): 2026/01/24

Reporting Summary

Nature Portfolio wishes to improve the reproducibility of the work that we publish. This form provides structure for consistency and transparency in reporting. For further information on Nature Portfolio policies, see our Editorial Policies and the Editorial Policy Checklist.

Statistics

For all statistical analyses, confirm that the following items are present in the figure legend, table legend, main text, or Methods section.

n/a	Confirmed	
☐	☒	The exact sample size (*n*) for each experimental group/condition, given as a discrete number and unit of measurement
☐	☒	A statement on whether measurements were taken from distinct samples or whether the same sample was measured repeatedly
☐	☒	The statistical test(s) used AND whether they are one- or two-sided<br>*Only common tests should be described solely by name; describe more complex techniques in the Methods section.*
☐	☒	A description of all covariates tested
☐	☒	A description of any assumptions or corrections, such as tests of normality and adjustment for multiple comparisons
☐	☒	A full description of the statistical parameters including central tendency (e.g. means) or other basic estimates (e.g. regression coefficient) AND variation (e.g. standard deviation) or associated estimates of uncertainty (e.g. confidence intervals)
☐	☒	For null hypothesis testing, the test statistic (e.g. F, t, r) with confidence intervals, effect sizes, degrees of freedom and P value noted<br>*Give P values as exact values whenever suitable.*
☒	☐	For Bayesian analysis, information on the choice of priors and Markov chain Monte Carlo settings
☒	☐	For hierarchical and complex designs, identification of the appropriate level for tests and full reporting of outcomes
☐	☒	Estimates of effect sizes (e.g. Cohen's *d*, Pearson's *r*), indicating how they were calculated

Our web collection on statistics for biologists contains articles on many of the points above.

Software and code

Policy information about availability of computer code

Data collection	Biorad ImageLab was used to image all Western Blots. LAS X software from Leica was used to acquire all immunohistochemistry, thioflavin and DiI staining images. ANY-maze was used to track mouse behavior during the Morris Water Maze test. Agilent software was used for GC/MS experiments. pClamp11 was used to acquire electrophysiology data.
Data analysis	Image J was used to analyze all Western Blotting images, all immunohistochemistry, thioflavin and DiI staining images. ANY-maze was used to analyze mouse behavior during the Morris Water Maze test. Agilent MassHunter software was used to analyze GC/MS experiments. Clampfit 11 software was used to analyze electrophysiology data.

For manuscripts utilizing custom algorithms or software that are central to the research but not yet described in published literature, software must be made available to editors and reviewers. We strongly encourage code deposition in a community repository (e.g. GitHub). See the Nature Portfolio guidelines for submitting code & software for further information.

Data

Policy information about availability of data

All manuscripts must include a data availability statement. This statement should provide the following information, where applicable:

- Accession codes, unique identifiers, or web links for publicly available datasets
- A description of any restrictions on data availability
- For clinical datasets or third party data, please ensure that the statement adheres to our policy

All data generated or analyzed during this study are included in this published article (and its supplementary information files).

Research involving human participants, their data, or biological material

Policy information about studies with human participants or human data. See also policy information about sex, gender (identity/presentation), and sexual orientation and race, ethnicity and racism.

Reporting on sex and gender	No human participants or data
Reporting on race, ethnicity, or other socially relevant groupings	NA
Population characteristics	NA
Recruitment	NA
Ethics oversight	NA

Note that full information on the approval of the study protocol must also be provided in the manuscript.

Field-specific reporting

Please select the one below that is the best fit for your research. If you are not sure, read the appropriate sections before making your selection.

☒ Life sciences ☐ Behavioural & social sciences ☐ Ecological, evolutionary & environmental sciences

For a reference copy of the document with all sections, see nature.com/documents/nr-reporting-summary-flat.pdf

Life sciences study design

All studies must disclose on these points even when the disclosure is negative.

Sample size	No statistical methods were used to determine sample size. For biochemical characterization of PSD-95 levels and palmitoylation, we used 15 or 12 wells gels and thus ran 10-12 samples in each gel. To have sufficient samples, we only compared two conditions in each experiment. For behavioral experiments, we consulted with Dr. Robert Rissman, an expert in using AD mouse models, and decided on an N=10-15. Immunohistochemistry, thioflavin staining and metabolomic data comes from animals used in behavioral experiments. For electrophysiology and spine analysis shown in Figure 5, we determined the required sample size from published similar studies.
Data exclusions	For the Morris Water Maze experiments, we excluded mice that did not learn to find the platform during the training phase. Mice were excluded if their average latency to platform was higher than 75s on days 3-7 of training and/or the total distance traveled was higher than 15 meters. Only 0-2 mice per group were excluded that way. For electrophysiology, recordings with Ra larger than 30 MΩ or with variations larger than 20% were excluded from data analysis. During analysis, events with absolute value of peak amplitude smaller than 4 pA were excluded from quantification.<br>In all other datasets, no data was excluded. This information is included in the Methods section of our manuscript.
Replication	We made sure that our data was reproducible by conducting experiments in several number of animals. We also conducted the immunohistochemistry and Western Blotting experiments several times and obtained similar results. A representative experiment is shown for all data presented.
Randomization	Mice were randomly selected for the treatment (Palm B injections) or the control (vehicle) groups
Blinding	All data was analyzed blind to the experimental condition.

Reporting for specific materials, systems and methods

We require information from authors about some types of materials, experimental systems and methods used in many studies. Here, indicate whether each material, system or method listed is relevant to your study. If you are not sure if a list item applies to your research, read the appropriate section before selecting a response.

Materials & experimental systems

n/a	Involved in the study
☐	☒ Antibodies
☒	☐ Eukaryotic cell lines
☒	☐ Palaeontology and archaeology
☐	☒ Animals and other organisms
☒	☐ Clinical data
☒	☐ Dual use research of concern
☒	☐ Plants

Methods

n/a	Involved in the study
☒	☐ ChIP-seq
☒	☐ Flow cytometry
☒	☐ MRI-based neuroimaging

Antibodies

Antibodies used

For immunohistochemistry, we used: human anti-PF11 (#AG-27B-0021-C100, AdipoGen), rabbit anti-PSD-95 (#51-6900, Invitrogen), chicken anti-MAP2 (#CPCA-MAP2, EnCor), rabbit anti-GFAP (#MAB360, Sigma Aldrich), rabbit anti-GluA3 (#AGC-010, Alomone labs). For Western Blotting, we used: mouse PSD-95 (#MA1-045, Thermo-Fisher) and mouse β-actin (#3700, Cell Signaling).

Validation

PF11 and PSD-95 (both the #51-6900 and the #MA1-045) primary antibodies were validated in PSD-95 Knock-out mice, see Figure S3. Link to CiteAB references for MAP2 antibody, cited 105 times: https://www.citeab.com/antibodies/1201449-cpca-map2-chicken-polyclonal-antibody-to-map2a-b
For the GFAP antibody, the supplier mentions that quality is routinely evaluated by Western Blot on Mouse brain lysates. This antibody also has numerous citations. The GluA3 antibody was used for immunofluorescence in these two publications: Parkinson, G.T. et al. (2018) Sci. Rep. 8, 4155. And Hamad, M.I. et al. (2011) Development 138, 4301. The β -actin antibody used has 5340 citations.

Animals and other research organisms

Policy information about studies involving animals; ARRIVE guidelines recommended for reporting animal research, and Sex and Gender in Research

Laboratory animals

Male and female APP/PS1 AD model mice (MMRRC Strain #034829-JAX) and their WT littermates aged between 9 and 10 months were used for all experiments. Mice were bred with C57BL/6J (#000664-JAX) in order to maintain a live colony.

Wild animals

n/a

Reporting on sex

Male and female mice were used, data is clearly presented and reports the sex of the mice used in all experiments.

Field-collected samples

n/a

Ethics oversight

This statement will be added at the end of the 'Mice' section of our manuscript Materials and Methods: All procedures involving animals were approved by the University of California San Diego Institutional Animal Care and Usage Committee (IACUC), and met the guidelines of the National Institute of Health detailed in the Guide for the Care and Use of Laboratory Animals.

Note that full information on the approval of the study protocol must also be provided in the manuscript.

Plants

Seed stocks

NA

Novel plant genotypes

NA

Authentication

NA
